# Bioluminescent Sensors for Ca^++^ Flux Imaging and the Introduction of a New Intensity-Based Ca^++^ Sensor

**DOI:** 10.3389/fbioe.2021.773353

**Published:** 2021-10-27

**Authors:** Jie Yang, Carl Hirschie Johnson

**Affiliations:** ^1^ Britton Chance Center for Biomedical Photonics, Wuhan National Laboratory for Optoelectronics-Huazhong University of Science and Technology, Wuhan, China; ^2^ Department of Biological Sciences, Vanderbilt University, Nashville, TN, United States

**Keywords:** GECIs, bioluminescent imaging, nanoluc, luciferin, real-time imaging

## Abstract

Sensitive detection of biological events is a goal for the design and characterization of sensors that can be used *in vitro* and *in vivo*. One important second messenger is Ca^++^ which has been a focus of using genetically encoded Ca^++^ indicators (GECIs) within living cells or intact organisms *in vivo*. An ideal GECI would exhibit high signal intensity, excellent signal-to-noise ratio (SNR), rapid kinetics, a large dynamic range within relevant physiological conditions, and red-shifted emission. Most available GECIs are based on fluorescence, but bioluminescent GECIs have potential advantages in terms of avoiding tissue autofluorescence, phototoxicity, photobleaching, and spectral overlap, as well as enhancing SNR. Here, we summarize current progress in the development of bioluminescent GECIs and introduce a new and previously unpublished biosensor. Because these biosensors require a substrate, we also describe the pros and cons of various substrates used with these sensors. The novel GECI that is introduced here is called CalBiT, and it is a Ca^++^ indicator based on the functional complementation of NanoBiT which shows a high dynamic change in response to Ca^++^ fluxes. Here, we use CalBiT for the detection of Ca^++^ fluctuations in cultured cells, including its ability for real-time imaging in living cells.

## Introduction

Free calcium ions (Ca^++^) are an important secondary messenger in cells. Key physiological processes such as cellular differentiation (Machaca, 2007), proliferation (Capiod, 2011), signal transmission (Zurgil et al., 1986), and muscle contraction (Kuo and Ehrlich, 2015) require Ca^++^ signaling. In the past decades, various versions of genetically encoded Ca^++^ indicators (GECIs) have been developed for the detection of Ca^++^ fluxes in cells (Greenwald et al., 2018). A GECI is an artificially designed molecule that converts chemical signals in the form of Ca^++^ concentration into optical signals and allows the detection or visualization of Ca^++^ fluctuations in a real-time, dynamic, and noninvasive manner in living cells and tissues.

Two types of GECIs involve changes in fluorescence or bioluminescence properties in response to changes in Ca^++^ levels (Greenwald et al., 2018). Fluorescent GECIs are excited by light, after which time they emit photons. On the other hand, bioluminescent GECIs do not require excitation by light, but instead require a substrate for chemical conversion and subsequent release of photons. The substrates are called “luciferins” and the enzymes that accomplish this bioluminescence reaction are called “luciferases;” most luciferases require the presence of oxygen to allow the bioluminescence reaction (Wilson and Hastings, 2013). Compared with bioluminescent GECIs, fluorescent GECIs can often be made to produce relatively stronger signals because the intensity of the excitation can be increased [with concomitant deleterious consequences of the strong irradiation (Robertson et al., 2013)]. However, because bioluminescent GECIs do not require excitation light, they have advantages, including no tissue autofluorescence, phototoxicity, photobleaching, or spectral overlap between sensor and optogenetic actuators. Regarding the latter point, bioluminescent GECI sensors can couple optimally with optogenetic probes as they do not require light excitation, and therefore light excitation can be used exclusively to active the optogenetic actuator without spectral crosstalk to the sensor (Yang et al., 2016). In contrast, because continuous excitation is required for the stimulation of fluorescent GECIs, combining these fluorescent sensors with optogenetic probes requires very careful separation of spectral overlaps, which can never be completely eliminated. Finally, owing to the low background, bioluminescent GECIs also have a very high signal-to-noise ratio (SNR) and are suitable for less-invasive deep tissue *in-vivo* imaging in live animals.

Herein we discuss the currently available bioluminescent GECIs, as well as introducing a novel bioluminescent GECI that is based upon functional complementation of a luciferase that is mediated by changes in Ca^++^ levels.

## Materials and Methods

### Protein Purification and *in vitro* Experiments

To purify CalBiT fusion proteins, we expressed each CalBiT in a modified bacterial expression vector pRSETb. The cDNA sequences were inserted between the *EcoR*I and *Hind*III restriction enzyme sites of pRSETb. The plasmid was transformed into BL21(DE3) *Escherichia coli* cells for the expression and purification of the fusion protein. The His6-tagged CalBiT proteins were purified using TALON Metal Affinity Co^++^ Resin. The signal intensity of each purified protein in response to varying [Ca^++^] was measured using Ca^++^ buffers (Molecular Probes, Life Technologies) and a fluorescence spectrophotometer (QuantaMaster, Photon Technology International Inc). The free Ca^++^ buffers of varying concentrations were prepared according to the manufacturer’s protocol. Briefly, buffer 1 (10 mM EGTA, 100 mM KCl, and 30 mM MOPS, pH 7.2) and buffer 2 (10 mM CaEGTA, 100 mM KCl, and 30 mM MOPS, pH 7.2) were mixed in different proportions to prepare varying free Ca^++^ concentrations. For all *in vitro* experiments, 10 μM final concentration of the NLuc substrate furimazine was added.

### Cellular Expression and Characterization

To construct the CalBiT1.0-3.0 family, we used the 11S (residues 1–156) and 114 (residues 157–169) versions of LgBiT and SmBiT, respectively (Dixon et al., 2016). The CalBiT2.0 sequence was constructed using a plasmid with the CAG promoter (pCAG) (Niwa et al., 1991), and CalBiT2.0 was expressed under the control of pCAG when transfected via Lipofectamine 2000 (ThermoFisher Scientific Inc.) into either HEK293 or HeLa cells grown in Dulbecco’s Modified Eagle Medium (Gibco). Two days later, signal responses were recorded using an inverted Olympus IX-71 epi-fluorescence microscope inside a temperature-controlled, light-tight box with a cooled Electron Multiplying-CCD (EM-CCD) camera. During real-time imaging, changes in cytosolic Ca^++^ were elicited by the addition of 10 μM histamine to HeLa cells (Figures 4A,B) or thapsigargin to HEK cells (Figure 4C), and the intensity of CalBiT2.0 luminescence was assayed. Expression of CalBiT2.0 was coupled with expression of the optogenetic melanopsion (*Opn4*) by constructing a bi-cistronic expression plasmid with a T2A coding sequence using CalBiT2.0 plus mouse melanopsin (*Opn4*) under the control of pCAG. The cells were transfected with the CalBiT2.0–2A-Opn4 construct and stimulated with a blue light pulse (470 ± 30 nm) for 1 s and later with 1 μM thapsigargin (Figure 4D).

### Data Analyses

**FIGURE 1:**
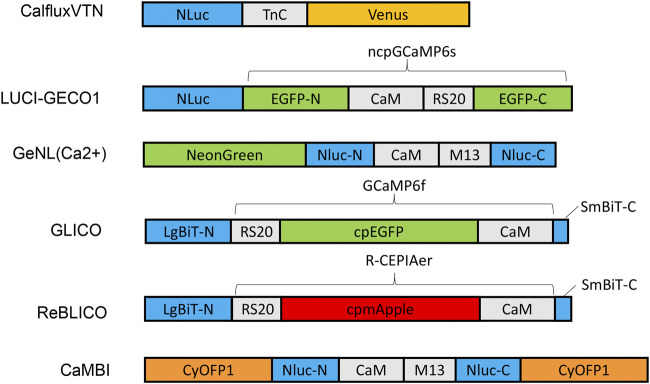
Schematic diagram of various designs and constructs of bioluminescent GECIs based on NLuc or NanoBiT. Abbreviations: LgBiT-N, N-terminal large NanoBiT; SmBiT-C, C-terminal small NanoBiT; CaM, calmodulin; RS20 and M13, calmodulin-binding peptide; NLuc-N and NLuc-C, N-terminal and C-terminal of NanoLuc, respectively.

The average light intensity in the regions of interest within cells was analyzed using ImageJ software (NIH). The Hill coefficient and Kd values (Figure 1C) were determined using the OriginLab 6 software (OriginLab).

## Review of Currently Available Bioluminescent GECIs

### Sensitive Imaging Using Bioluminescent GECIs

A sensitive GECI requires high signal intensity, a large dynamic range, and rapid kinetics. However, bioluminescent sensors as a general observation generate relatively weaker signal intensity and therefore imaging usually requires a sensitive charge-coupled device (CCD) camera with a relatively longer exposure time so as to capture more photons, thereby attaining sufficient resolution. Some physiological reactions are involved in fast kinetic events of Ca^++^ flux in cells, for example, the action potentials of excitatory neurons with millisecond-level responses (Müller et al., 2007). For imaging such rapid events, brief exposures are necessary. In such cases, bright sensors are required to obtain enough photons within brief exposures for acquiring sufficient spatial and temporal resolution for the resulting images. To improve the signal intensity of bioluminescent molecules, the following two strategies are usually adopted: 1) development and use of brighter bioluminescent systems based on brighter luciferases and/or luciferins. For instance, bioluminescent systems have been developed from the first generation aequorin to the brighter third-generation engineered luciferase called NanoLuc (NLuc) (Hall et al., 2012); 2) to increase the intensity by intramolecular bioluminescence resonance energy transfer (BRET) between a luciferase and a fluorophore (Takai et al., 2015) (see discussion below in part 3). The following two reasons may explain why the signal intensity can sometimes be enhanced by BRET: 1) luciferase enzyme sometimes forms a relatively more stable molecular conformation when fused with a fluorescent protein (Baubet et al., 2000), and/or 2) when a fluorescent protein with high extinction coefficient and quantum yield acts as a BRET acceptor, more efficient emission can be produced via non-radiative energy transfer (Saito et al., 2012).

Apart from signal intensity, affinity and dynamic range are two key factors for sensitive imaging using bioluminescent GECIs (Chiu and Haynes, 1977; Pérez Koldenkova and Nagai, 2013). The dissociation constant (Kd) stands for the binding capacity between the indicating sensor and Ca^++^, and it can be determined using buffers prepared with ethylene glycol tetraacetic acid (EGTA) and Ca^++^-EGTA to achieve specific concentrations of free calcium ions (Pérez Koldenkova and Nagai, 2013; Yang et al., 2016). These data are normalized to the maximal (defined as 1.0 under Ca^++^ saturation conditions) and to the minimal (defined as 0 under Ca^++^ depletion conditions) signal intensity or BRET ratio value. The dynamic range is defined as the maximal intensity or BRET ratio (in a saturated Ca^++^-bound state) divided by the minimal intensity or BRET ratio (determined in the presence of zero-added calcium plus EGTA). In living cells, when resting Ca^++^ concentration lies within the detectable range of the indicator/sensor, high baseline signals can be generated using a high affinity (low Kd value) indicator. Similarly to the measurement of different lengths where a micrometer is optimal for micrometers to a centimeter while a meter-stick is best for lengths between a centimeter and a meter, measuring intracellular Ca^++^ levels requires the selection of the most appropriate measuring sensor. As different cell organelles contain different Ca^++^ concentrations, it is necessary to develop GECIs with appropriate Kd values (e.g., higher Kd for high Ca^++^ regions, etc.). To address this need, Hossain and co-workers developed a family of multicolor bioluminescent GECIs with different Kd values for simultaneous imaging in the endoplasmic reticulum (ER), nucleus, and mitochondria (Hossain et al., 2018).

The detection methods of GECIs include intensiometric and ratiometric imaging (Greenwald et al., 2018). Intensiometric imaging assesses in the change of signal intensity in response to changes in Ca^++^ levels and is effectively using a single wavelength channel. On the other hand, ratiometric imaging requires the calculation of the ratio between the intensities at two (or more) different wavelengths and the data are therefore collected from two (or more) channels. The intensiometric method is relatively easier to perform, but as its results are obtained by a single channel, they can be disturbed by movement artifacts, out-of-focus, changes in the level of expression of the sensor, and decay of the active substrate concentration during a long imaging interval. Any of those effects can cause serious artifacts and therefore incorrect conclusions. In contrast, ratiometric imaging corrects the influences of those adverse factors, but requires dual-view dichroic equipment to split the image into light of different contributing wavelengths, such as W-VIEW GEMINI 2C Image Splitting Optics (https://www.micromecanique.fr/product/view/fc0ff0d4–87d2–4a2f-b302–17dfb890f2fa). Consequently, while ratiometric imaging has many advantages, its need for an image splitting system increases the experimental cost of the imaging system. Alternatively, some of the adverse factors can be minimized for short-term recording session if an intensiometric indicator with a high enough dynamic range is used because the change from out-of-focus and decay of the substrate artifacts can be neglected when genuine signal changes from the GECI is large and the recording interval is short.

### Development of Bioluminescent GECIs

Three generations of bioluminescent GECIs based on different luciferases have been developed as of the present time (Table 1). Aequorin, the first generation bioluminescent GECI, was identified and isolated from jellyfish along with the green fluorescent protein (GFP) by Shimomura and colleagues (Shimomura et al., 1962). In native jellyfish, aequorin interacts closely with GFP to form a BRET molecular complex so that the intrinsically blue luminescence of aequorin is converted to green luminescence by virtue of Förster resonance energy transfer (Morin and Hastings, 1971). Aequorin contains three EF-hand domains of Ca^++^-binding sites, and exists at low Ca^++^ concentrations bound to the substrate/luciferin (coelenterazine) in an enzymatically locked intermediate form. The binding of Ca^++^ induces a conformational change that allows the completion of the enzymatic reaction, converting coelenterazine to coelenteramide and releasing a photon of blue light. Unlike Firefly luciferase (FLuc), the final step of the aequorin reaction is not dependent upon ATP, and therefore its reaction is allowed in ATP-deficient domains, e.g., extracellular spaces (Montero et al., 2000).

**TABLE 1:** Summary of properties of bioluminescent GECIs.

GECI names	Luciferase	Substrate	Readout	Kd (μM)	References
Aequorin	Aequorin	Coelenterazine	Intensity	2.6	Tricoire et al. (2006)
GFP-Aequorin	Aequorin	Coelenterazine	Intensity	Not determined	Baubet et al. (2000)
BRAC	Rluc8	Coelenterazine	Ratio	1.9	Saito et al. (2010)
Nano-lantern (Ca2+)	Split Rluc8	Coelenterazine	Intensity	0.62	Saito et al. (2012)
CalfluxVTN	NLuc	Furimazine	Ratio	0.48	Yang et al. (2016)
LUCI-GECO1	NLuc	Furimazine	Ratio	0.285	Qian et al. (2019)
GeNL (Ca2+)	Split NLuc	Furimazine	Intensity	Four versions: 0.060; 0.25; 0.48; 0.52	Suzuki et al. (2016)
GLICO	NanoBiT	Furimazine	Intensity	0.23	Farhana et al. (2019)
ReBLICO	NanoBiT	Furimazine	Intensity	1,526	Farhana et al. (2019)
Orange CaMBI	Split NLuc	Furimazine; Hydrofurimazine	Intensity	Four versions: 0.11; 0.2; 0.27; 0.3	Oh et al. (2019)

Aequorin is useful for monitoring Ca^++^ concentration in cells or tissues, and it responds to increases in Ca^++^ with flashes that discharge the aequorin; the inactive apo-aequorin can be recharged to active aequorin, but the recharging process can require hours. Moreover, the bioluminescent flashes of aequorin are relatively dim. To enhance the intensity of the aequorin signal, Baubet and co-workers fused GFP onto apo-aequorin to form an intramolecular BRET indicator, known as GFP-Aequorin, which increased the signal intensity by 16-fold to 65-fold (Baubet et al., 2000). It is not clear whether this enhancement is due to an increase in the intensity of each flash, or if the turnover of GFP-Aequorin is slower than that of aequorin, and therefore larger amounts of GFP-Aequorin accumulate in cells as a result of expression of the transgene. GFP-Aequorin has been used to monitor Ca^++^ concentration in different subcellular locations such as nucleus (Brini et al., 1993), mitochondria (Filippin et al., 2005), ER (Kendall et al., 1992), and the Golgi apparatus (Rodriguez-Garcia et al., 2014). To monitor neuronal activity in freely-behaving zebrafish, Naumann and co-workers used GFP-Aequorin to label ∼20 neurons of the hypocretin-positive hypothalamus in freely behaving zebrafish using a non-imaging approach and found that the neurons were associated with increased locomotor activity and identified two classes of neural activity corresponding to distinct swimming behaviors (Naumann et al., 2010).

GFP-Aequorin allows monitoring of Ca^++^ fluxes in cultured cells or living animals, but it still underperforms because of low brightness and slow recharge. Saito et al. developed two second-generation Ca^++^ sensors based on the continuously turning-over luciferase from *Renilla* (RLuc), e.g., BRAC (Saito et al., 2010) and Nano-Lantern(Ca^++^) (Saito et al., 2012); the particular version of RLuc was “RLuc8,” a bright mutant RLuc derived from consensus-guided mutagenesis (Loening et al., 2006). The ratiometric sensor BRAC is a fusion of Venus-CaM-M13-RLuc8 and it responds to binding of Ca^++^ to the calmodulin (CaM) moiety by intramolecular BRET between the RLuc8 and Venus; however, this ratiometric change has only a 0.6-fold dynamic range (Saito et al., 2010). Later, the same research group developed a non-ratiometric Ca^++^ sensor GECI based on Nano-Lantern (Saito et al., 2012). Nano-Lantern is a direct fusion of RLuc8 to Venus, and it showed a 10× higher luminescence intensity than that of RLuc8 alone. The Ca^++^ sensor Nano-Lantern(Ca^++^) was constructed by insertion of the Ca^++^-sensing domain CaM-M13 into the RLuc8 portion of Nano-lantern. Binding of Ca^++^ to CaM induces a conformational change that allows the inactive split RLuc8 to recombine and reconstitute its activity. Nano-lantern(Ca^++^) showed a great improvement in signal intensity compared with that of aequorin and enabled visualization of biological phenomena that could not be visualized with aequorin. Moreover, both BRAC and Nano-lantern(Ca^++^) rely on continuously turning-over luciferase that does not entail the slow recharging phenomenon that aequorin requires. However, these second-generation sensors still have weaker signal intensity than competing fluorescent sensors. For instance, Nano-Lantern(Ca^++^) luminescence still requires long (100 ms) exposure times, and is kinetically too slow for imaging fast events such as neuronal spikes (Saito et al., 2012).

The recent development and directed evolution of NLuc initiated a third generation of sensors that made the first two generations of sensors obsolete. The NanoLuc “system” was derived from bio-engineering to optimize both a luciferase and its luciferin to achieve more efficient light emission (Hall et al., 2012). Using a small but active fragment (19 kDa) of the much larger luciferase of the deep-sea shrimp *Oplophorus gracilirostris* (106 kDa), Hall and co-workers optimized the protein structure of the luciferase as well as testing analogs of the original luciferin (coelenterazine) to discover a novel imidazopyrazinone substrate (furimazine); the end result being a luciferase/luciferin combination with a specific activity 150-fold greater than that of either FLuc or RLuc systems similarly configured for glow-type assays. The final size of Nluc (19 kD) is approximately one-half the size of Rluc8 (36 kD) (Hall et al., 2012); small size is a useful characteristic when fusing a sensor to other proteins. Moreover, NLuc has a fast turnover rate of 6.6 reactions per second per molecule, which is 1.7-fold and 8,700-fold higher than that of RLuc8 and aequorin, respectively (Oh et al., 2019). Similarly to aequorin and Rluc, the NLuc reaction is not ATP-dependent.

Various GECI constructs based on NLuc have been developed. Ratiometric indicators such as CalfluxVTN (Yang et al., 2016) and LUCI-GECO1 (Qian et al., 2019) have been developed to detect Ca^++^ fluxes, e.g., in neurons and in conjuction with optogenetics. The ratiometric indicator, CalfluxVTN, is a fusion of three separate moieties: Venus, Troponin, and NLuc (Figure 1). CalfluxVTN exhibits strong Förster resonance energy transfer from NLuc to Venus under high Ca^++^ conditions (due to a conformational change mediated by binding of Ca^++^ to the Ca^++^-sensitive Troponin moiety), whereas the energy transfer almost disappears under low Ca^++^ conditions. The ratio of Venus to NLuc exhibits a large dynamic range (∼11 fold *in vitro* and 4∼6-fold dynamic change in cells) (Yang et al., 2016). The design of LUCI-GECO1 is different from that of CalfluxVTN, and is constructed by the fusion of NLuc with ncpGCaMP6s, a topological variant of GCaMP6s, to form an intramolecular BRET pair (Figure 1). Both CalfluxVTN and LUCI-GECO1 have fluorescent domains that can be helpful for tracking their localization or abundance independently of luminescence (the Venus moiety for CalfluxVTN, the ncpGCaMP acceptor domain for LUCI-GECO1) (Qian et al., 2019). Both CalfluxVTN and LUCI-GECO1 enable sensitive imaging of Ca^++^ fluxes under the stimulation of optogenetically induced Ca^++^ concentration changes in cultured neurons.

Intensiometric GECIs based on NLuc include GeNL (Ca^++^) (Suzuki et al., 2016), GLICO & ReBLICO (Farhana et al., 2019), and CaMBI (Oh et al., 2019) (Figure 1). Suzuki and co-workers developed GeNL(Ca^++^) based on GeNL, which is a BRET molecule formed by the fusion of NLuc and mNeonGreen, the brightest green fluorescent protein, resulting in ∼2-fold improvement of the emission signal intensity. The Ca^++^-sensitive CaM-M13 domain was inserted between Gly66 and Leu67 of the NLuc moiety. The GeNL-based Ca^++^ indicator exhibited ∼5-fold *in-vitro* and 1∼2-fold dynamic change in response to Ca^++^ changes in cultured cells (Suzuki et al., 2016). Nagai and co-workers developed GLICO and ReBLICO (Figure 1) (Farhana et al., 2019), which utilizes a binary complementation reporter NanoBiT system derived from Nluc (Dixon et al., 2016). GLICO is constructed by the fusion of two fragment components of NanoBiT, large BiT (LgBiT, 18 kDa) and small BiT (SmBiT, 1.3 kDa), with GCaMP6f. Similarly, ReBLICO is constructed by the fusion of LgBiT/R-CEPIA1er/SmBiT. Both GLICO and ReBLICO possess the advantages of fluorescent and bioluminescent GECIs with a wide range of applications (Farhana et al., 2019).

For *in-vivo* imaging deeper into a tissue than just the surface, high penetration of photons is required (Men and Yuan, 2019). Moreover, bioluminescence has a theoretical advantage over fluorescence for imaging tissues because only the emission light needs to penetrate the tissues, whereas for fluorescence it is a “two-way street” and penetration of the excitatory irradiation is also critical. Furthermore, red-shifted light, including near-infrared light, has better penetration power in tissue than blue-shifted light. However, the emission spectra of most GECIs range between blue and yellow, and therefore there is strong absorption of the light by the tissues–hence, poor penetration. Chu et al. developed a BRET reporter designated as Antares by the fusion of CyOFP1-NLuc-CyOFP1, in which CyOFP1 is a fluorescent protein with a large Stokes shift (in this case, an excitation {EX} peak at 470 nm and an emission {EM} peak at 570 nm) that emits orange-red light (Chu et al., 2016). Based on Antares, Oh and co-workers developed an orange GECI known as Orange CAMBI by the insertion of the CaM-M13 sequence into the Leu133 site of Antares NLuc (Figure 1) (Oh et al., 2019). Orange CaMBI was used to monitor Ca^++^ fluctuations in living mouse liver, wherein the liver lobes showed regionally differing phases of Ca^++^ oscillations (Oh et al., 2019). The 580–600 nm EM peak of Orange CaMBI is still far from the ideal optical window (750–1,000 nm). In future studies, the development of brighter and more red-shifted GECIs will be helpful for sensitive detection of Ca^++^ fluctuations within deep tissues *in vivo*.

### Current Commercially Available Substrates

Bioluminescence is an enzyme-catalyzed chemiluminescence reaction with a substrate in which the energy released is used to produce an intermediate in an electronically excited state, P*, which then emits a photon. The emission does not come from or depend on light absorbed, as in fluorescence, but the excited state produced is indistinguishable from that produced in fluorescence after the fluorophore has absorbed a photon. As such, the luciferin substrate is an essential partner with the luciferase in determining the characteristics of the overall luminescent system. The bioluminescence substrates D-luciferin and coelenterazine are naturally synthesized via biosynthetic pathways. D-luciferin is the most commonly used substrate of FLuc. The FLuc/D-luciferin system and its derivative series, including Fluc/CycLuc1 (Evans et al., 2014) and AKaLuc/AkaLumine-HCl (Kuchimaru et al., 2016; Iwano et al., 2018), require ATP and Mg^++^, which can fluctuate in a circadian manner (Feeney et al., 2016). So far, a few GECIs have been constructed on the basis of FLuc luminescence with D-luciferin or its analogs. However, most bioluminescent GECIs have been constructed using luciferases that catalyze reactions involving coelenterazine or its analogs such as native coelenterazine, coelenterazine-h coelenterazine-400a, bis-coelenterazine, Furimazine (Inouye et al., 2013), and 8pyDTZ (Yeh et al., 2019). Although D-luciferin is a more stable substrate than coelenterazine, most GECIs are based on coelenterazine-catalyzing luciferases because of other advantageous characteristics (e.g., brightness, wavelength of emission, insensitivity of the emission wavelength to pH, size of the luciferases, etc.).

An ideal luminescent substrate would be non-toxic, cell permeable, and stable (long half-life). Native coelenterazine and most of its analogs are hydrophobic and consequently freely permeable through the cell membrane. Also, native coelenterazine is not toxic, but on the other hand in the presence of oxygen, it auto-oxidizes and decays relatively quickly. Promeage Corporation developed two coelenterazine analogs, EnduRen™ and ViviRen™, that are two chemically modified substrates designed for the protection of oxidation sites in coelenterazine by esters or oxymethyl ethers (Otto-Duessel et al., 2006). Their protecting groups increase the half-life of these live-cell substrates in culture medium as compared with the unprotected coelenterazine substrates (including native coelenterazine). Because of the protecting groups, EnduRen™ and ViviRen™ cannot be oxidized by luciferases, but once they pass across plasma membranes and enter into viable cells, intracellular esterases and lipases cleave the protecting groups from the modified substrates, thus generating enzymatically activatable substrates (e.g., coelenterazine-h) that can be catalyzed by intracellular luciferases to emit light. Another benefit of using these protected substrates is that they enable a low background of auto-luminescence/oxidation in the extracellular medium because of the low concentration of active esterases and lipases in the culture medium; thus, the background signal can be decreased by 10- to 100-fold, thereby further improving the SNR (Otto-Duessel et al., 2006).

Furimazine, a highly specific substrate for NLuc, is an artificially synthesized coelenterazine analog which allows the NLuc/furimazine system that is 150× brighter than that of the Fluc/D-luciferin system (Hall et al., 2012). As mentioned earlier, third generation bioluminescent GECIs were developed based on NLuc/furimazine. However, the NLuc/furimazine system typically emits blue photons that are not optimal for *in vivo* applications that require tissue penetration. Yeh and co-workers optimized teLuc (NanoLuc-D19S/D85N/C164H)/diphenylterazine from NLuc and fused it to CyOFP1 (Yeh et al., 2017). Together with another coelenterazine analog (DTZ), the CyOFP1-teLuc-CyOFP1 combination enabled a new bioluminescence system in which the EM has two prominent peaks, one at 502 nm and the other at 580 nm, which is dramatically red-shifted from the standard NLuc/furimazine system (Yeh et al., 2017). Besides the emission of blue light, another characteristic of the NLuc/furimazine system that can be problematic in some applications is that furimazine is so hydrophobic that it exhibits poor solubility in some media (it is a tradeoff: this characteristic is good for membrane permeability, but can be troublesome for substrate application). To improve the solubility and bioavailability of the NLuc substrate in media, Su and co-workers developed a new substrate, hydrofurimazine (Su et al., 2020), that has enhanced aqueous solubility, thereby facilitating higher dose delivery to mice. In the mouse liver, hydrofurimazine with Antares (the fusion of CyOFP1-NLuc-CyOFP1) exhibited similar brightness as exhibited by the AkaLuc/AkaLumine system, thus allowing two-population imaging with these two luciferase systems.

## Results

### A New Functional Complementation/Intensity Sensor: CalBit

**FIGURE 2:**
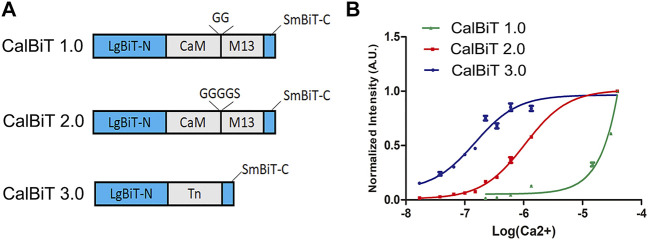
Characteristics of CalBiTs *in vitro*. **(A)** The CaM-M13 or troponin C domain (TnC) was inserted between LgBit-N and SmBiT-C, two functional complementation fragments of NanoBiT (Dixon et al., 2016). Three versions of CalBiT were developed using different linkers and Ca^++^-binding domains. In CalBiT1.0, two Gly residues were used as linkers between CaM and M13 and GGGGS was used as a linker between CaM and M13 in CalBiT2.0, whereas TnC was the Ca^++^-binding domain inserted between LgBit-N and SmBiT-C in CalBiT3.0. **(B)** Comparison of [Ca^++^] dependency *in vitro* for CalBiT1.0 (green line), CalBiT2.0 (red line), and CalBiT3.0 (blue line). The intensities of the three CalBiTs were measured by a QuantaMaster spectrofluorimeter (not using fluorescence excitation). The plotted values were normalized to the maximal signal intensity achieved at 39 mM Ca^++^. Values for the Hill coefficient and Kd of Calflux VTN are shown in Table 2, mean ± standard deviations. *n* = 3.

In addition to the foregoing summary of bioluminescent GECIs, we also introduce here a novel bioluminescent GECI based on the functional complementation of NanoBiT. Two NanoBiT subunits, LgBiT (large fragment) and SmBiT (small fragment), have been previously optimized to interact with low affinity and reversibly to reconstitute luminescence activity (Dixon et al., 2016). We used the NanoBiT concept to construct a novel GECI by sandwiching CaM-M13 between the LgBiT (18 kDa) and SmBiT (1.3 kDa) subunits of NLuc (we used the 11S {residues 1–156} and 114 {residues 157–169} versions of LgBiT and SmBiT, respectively). The conformational changes of CaM-M13 lead to reversible changes in the distance between the LgBit and SmBit subunits in response to Ca^++^ concentration, thereby leading to Ca^++^-dependent changes in luminescent intensity. We call these constructs that are based on NanoBiT “CalBiT”, and the schematic diagram of different versions of CalBiT is shown in Figure 2A. The CaM-M13 sequences are from the D3cpv plasmid, in which the CaM-M13 sequence has been altered into a version with low binding ability with native CaM but high affinity to the CaM sequence within the CaM-M13 cassette (Palmer et al., 2006). Therefore, the CaM-M13 of D3cpv will be minimally responsive to the Calmodulin protein that is endogenously present in cells.

To develop GECIs with appropriate affinity for Ca^++^, we used different linkers for the fusion of CaM and M13 (the linkers between CaM and M13 can modify the Ca^++^ binding affinity (Saito et al., 2012). We used combinations of different linkers such as GG (two Gly residues) and GGGGS (four Gly residues and one Ser residue) to construct two CaM-M13 versions of CalBiT, CalBiT1.0 and CalBiT2.0, which exhibit different Ca^++^ affinities, with 25 and 1.11 μM Kd values, respectively. We also created a version of CalBiT that is based on the Ca^++^-binding domain (TnC) domain of troponin C from *Opsanus tau*. This is the same Ca^++^-binding domain as that used in Twitch-3 and CalfluxVTN (Thestrup et al., 2014; Yang et al., 2016). We designated this TnC-based CalBiT as CalBiT3.0, and it showed a high Ca^++^ affinity with a Kd value of 0.14 μM.

**FIGURE 3:**
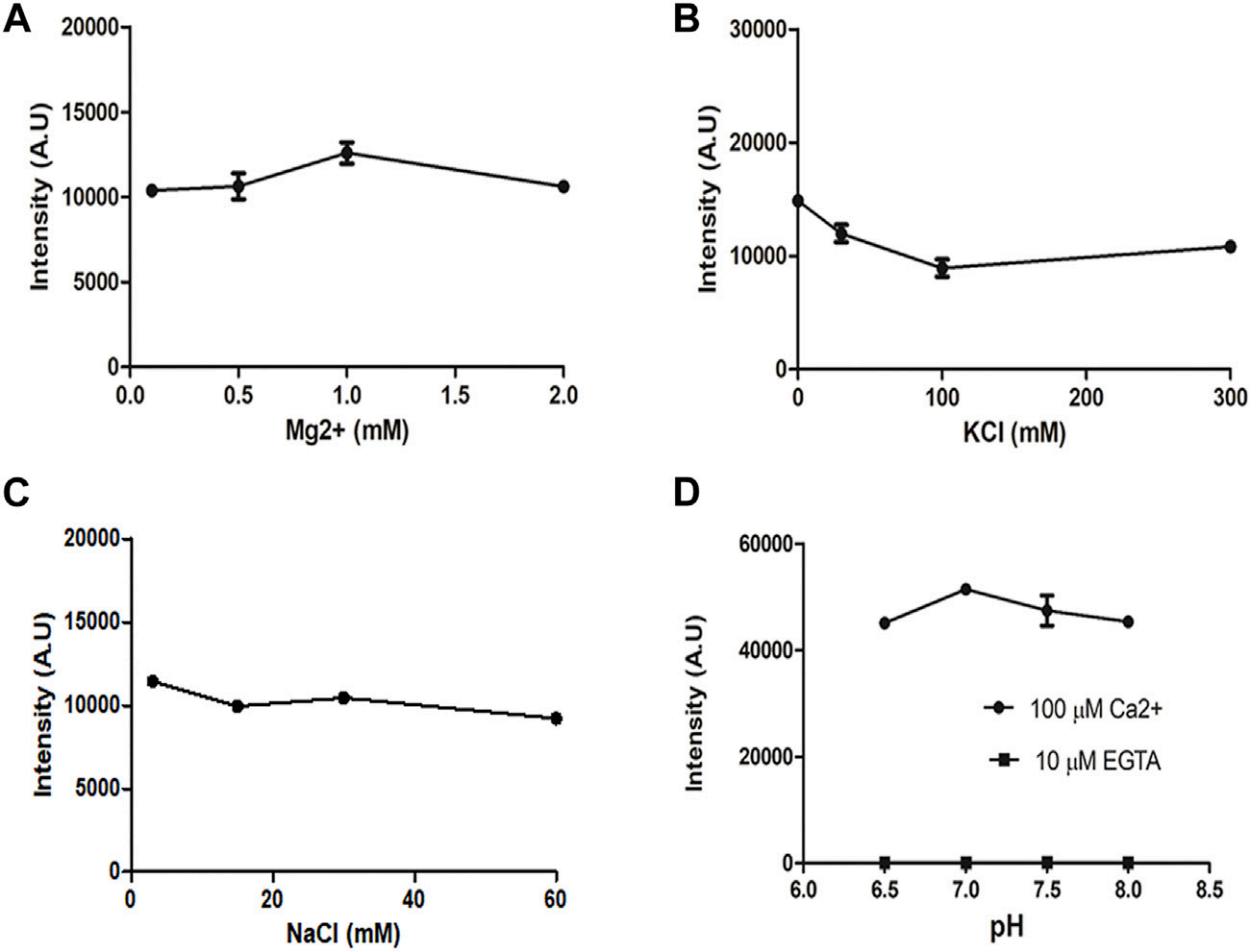
Insensitivity of CalBiT2.0 to Mg, K^+^, Na^+^, and H^+^ ions *in vitro*. CalBiT2.0 was purified using a 6-His tag and the intensity was recorded in solutions with increasing concentrations of the ions as shown. The data are plotted as intensity in response to increasing [Mg^++^] **(A)**; [K^+^] **(B)**; [Na^+^] **(C)**; H^+^ as pH **(D)** (mean ± standard deviations. *n* = 3).^++^.

CalBiT1.0 and CalBiT2.0 exhibited dynamic ranges of 67-fold and 56-fold, respectively (Figure 2B and Table 2). As per our knowledge, there are no reported GECIs that can exceed CalBiT1.0 and CalBiT2.0 in terms of dynamic range. The higher Kd of CalBiT1.0 may be useful for measuring Ca^++^ levels in subcompartments of cells that have higher resting Ca^++^ levels, such as the ER or mitochondria. CalBiT2.0 has a Kd value of 1.11 μM, and therefore of these three CalBiTs, it is the most suitable for the detection of Ca^++^ fluxes in the cytosolic compartment. The intensity of the CalBiT family is not sensitive to the changes in Mg^++^, K^+^, and Na^+^ concentrations or pH that might be expected to occur within the physiological ranges in cells; the properties for CalBiT2.0 are shown in Figure 3.

**TABLE 2:** Properties of three versions of CalBiT reported here.

	∆I/I (%)	Kd (μM)	Hill coefficient
CalBiT 1.0	67	25	2.22
CalBiT 2.0	56	1.11	1.49
CalBit 3.0	5.5	0.14	1.22

**FIGURE 4:**
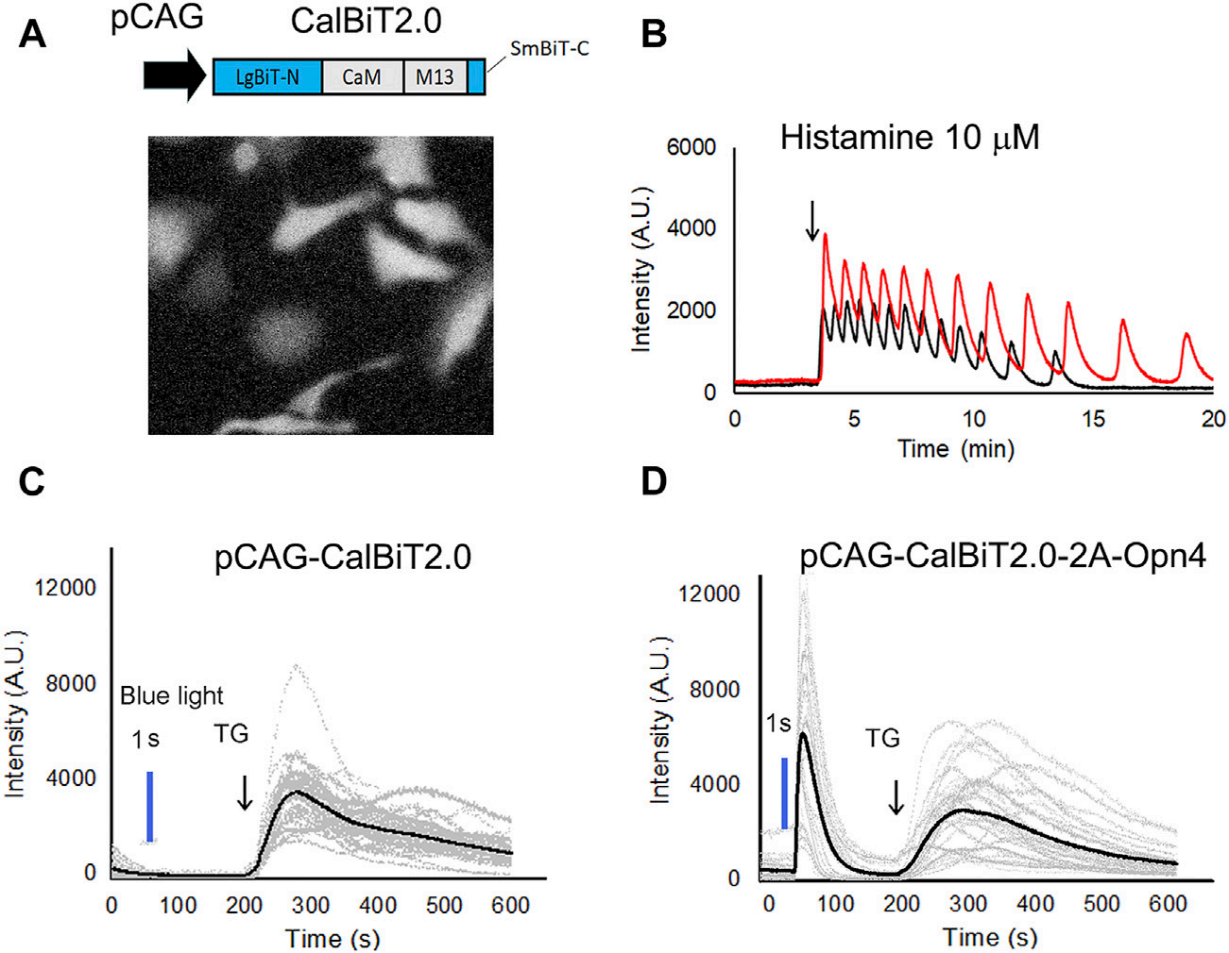
CalBiT2.0 as a Ca^++^ sensor in mammalian cells. **(A)** CalBiT2.0 construct driven by the CAG promoter (pCAG) for mammalian cell expression (upper), and snap-shot photomicrographs of HeLa cells expressing CalBiT2.0 during the addition of furimazine substrate and 10 μM histamine into media (below). **(B)** Two HeLa cells (red and black traces, respectively) transfected with the pCAG-CalBiT2.0 plasmid. The signal intensity of the two cells showed out-of-phase oscillations in response to 10 μM histamine. The arrow shows when histamine was added. See also Supplementary Movie S1. **(C)** and **(D)** Monitoring Ca^++^ fluxes with CalBiT2.0 in response to an optogenetic actuator (melanopsin encoded by *Opn4*) and/or an agent that releases store-operated Ca^++^ (thapsigargin, TG). HEK293 cells transfected with pCAG-driven construct encoding CalBiT2.0 alone **(C)** or transfected with bicistronic pCAG-driven construct encoding CalBiT2.0 and melanopsin (*Opn4*, **(D**). Both groups of cells in **(C and D)** were exposed to blue light (blue line) for 1 s, followed by the addition of 1 μM thapsigargin (at the time indicated by the arrow and “TG”). The gray lines represent the signal intensity for each cell as a function of time, and the black line represents the average signal intensity of all cells. HEK293 cells that do not express melanopsin (panel **C**) did not exhibit a Ca^++^ flux in response to the blue light pulse, but HEK293 cells that additionally express melanopsin (panel **D**) showed large Ca^++^ changes in response to optogenetic actuation. Both the groups of the HEK293 cells exhibit large and persisting changes in signal intensity in response to stimulation of store-operated Ca^++^ release by thapsigargin.

Histamine can stimulate Ca^++^ oscillations in HeLa cells (Sauvé et al., 1991). We expressed CalBiT2.0 in HeLa cells under the control of the CAG promoter, and used 10 μM histamine to stimulate the cells (Figure 4A). The CalBiT2.0 intensity signal reported robust histamine-induced Ca^++^ oscillations in the HeLa cells within a large amplitude (∼10-fold) following the addition of furimazine (substrate for CalBiT) and 10 μM histamine into the culture medium. Also, different cells oscillated independently, as indicated by the out-of-phase rhythms of the CalBiT signal (Figure 4B, also see Supplementary Movie S1).

Next, we investigated whether CalBiT2.0 can be coupled with an optogenetic probe. Since melanopsin triggers the release of internal calcium stores in response to blue light (Kumbalasiri, et al., 2007; Qiu et al., 2005), we further constructed a pCAG-CalBiT2.0–2A-Opn4 plasmid having a self-cleaving T2A sequence for the fusion of CalBiT2.0 with melanopsin (gene name is *Opn4*) and formation of a co-expression sequence. We transfected CalBiT2.0 into HEK293 cells with and without *Opn4* co-expression. Ca^++^ levels in HEK293 cells transfected with the pCAG-CalBiT2.0 plasmid increased in response to thapsigargin (TG) but not in response to blue light (Figure 4C). The cells with co-expression of CalBiT2.0 and melanopsin exhibited an increase in Ca^++^ levels in response to both thapsigargin and blue light (Figure 4D).

## Discussion

We summarize here the bioluminescent GECIs that are available for measuring Ca^++^ fluxes in cells. Primarily, we focus on the design and application of the third generation of bioluminescent GECIs based on NLuc which, because of their brightness, generally have an advantage for assaying rapid Ca^++^ response events with high signal-to-noise ratio (SNR) in cells, which is especially important for neurons. Although fluorescent GECIs are currently the most commonly used Ca^++^ sensors for neuron imaging in brains of living animals, however, the optic fibers that are required for those measurements (for both EX and EM) will inevitably damage the brain tissues and perturb authentic Ca^++^ responses. We believe that there is great promise toward the realization of non-invasive Ca^++^ imaging of neuronal activity without the use of the fiber optics as bioluminescent GECI technology continues to develop in the future. In particular, with genetic tools to express and target the sensor, and substrates that can cross the blood-brain barrier–in conjunction with a red-shifted EM, a completely non-invasive measurement of Ca^++^ fluxes in freely behaving animals can be accomplished. However, there are still some problems to be solved when using bioluminescent GECIs for non-invasive Ca^++^ imaging *in vivo*. For instance, the substrates need to be more compatible with tissues, especially we need to develop substrates that are permeable across the blood-brain barrier for neural imaging. A more water-soluble furimazine called hydrofurimazine has been reported to be feasible for liver tissue imaging after injection into the tail vein (Su et al., 2020), but whether it can effectively permeate across the blood brain barrier has not yet been verified. Furthermore, for deep tissue Ca^++^ imaging, the red-shifted Orange CaMBI has been developed to monitor Ca^++^ fluctuations in living animals (Oh et al., 2019). However, the 580–600 nm EM peak of Orange CaMBI is still far from the ideal far-red/infrared optical window (750–1,000 nm). We envisage the development of brighter and more red-shifted GECIs that will be optimal for sensitive detection of Ca^++^ fluxes within deep tissues *in vivo*.

We also introduce here a family of novel bioluminescent intensitometric GECIs. The calcium response range of CalBiT2.0 looks promising for monitoring physiological Ca^++^ changes in the cytosol. CalBiT2.0 exhibited large dynamic ranges *in vitro* (56-fold) and in living cells (∼10-fold) (Figures 2, 4). CalBiT2.0 effectively reported Ca^++^ fluxes resulting from Ca^++^ oscillations and optogenetic stimulation of melanopsin (Figure 4). Although the maximum signal intensity of CalBiT2.0 is weaker than that of CalfluxVTN (Yang et al., 2016), further optimization of CalBiT2.0 is warranted to create a next-generation sensor with the large dynamic range of CalBiT2.0 and a brighter signal.

## Data Availability

The original contributions presented in the study are included in the article/Supplementary Material, further inquiries can be directed to the corresponding author.
